# Selenium status in soil, water and essential crops of Iran

**DOI:** 10.1186/1735-2746-9-11

**Published:** 2012-11-21

**Authors:** Lyly Nazemi, Shahrokh Nazmara, Mohammad Reza Eshraghyan, Simin Nasseri, Kurosh Djafarian, Masoud Yunesian, Hassan Sereshti, Aziz Moameni, Seyed Jamaleddin Shahtaheri

**Affiliations:** 1 Department of Nutrition and Biochemistry, School of Public Health and Maternal, Fetal and Neonatal Research Center (MFNRC), Tehran University of Medical Sciences, Tehran, Iran; 2 Department of Environmental Health Engineering, School of Public Health, and Center for Water Quality Research (CWQR), Institute for Environmental Research (IER), Tehran University of Medical Sciences, Tehran, Iran; 3 Department of Epidemiology and Biostatistics, School of Public Health, Tehran University of Medical Sciences, Tehran, Iran; 4 School of Chemistry, College of Sciences, University of Tehran, Tehran, Iran; 5 Department of Occupational Health, Institute for Environmental Research (IER). School of Public Health and Center for Water Quality Research (CWQR), Tehran University of Medical Sciences, Tehran, Iran; 6 Soil and Water Research Institute, Tehran, Iran; 7 Department of Nutrition and Biochemistry, School of Public Health, Tehran University of Medical Sciences, Tehran, Iran

**Keywords:** Selenium, Agriculture, Soil, Water, Essential crops

## Abstract

**Abstracts:**

As a contributing factor to health, the trace element selenium (Se) is an essential nutrient of special interest for humans and all animals. It is estimated that 0.5 to 1 billion people worldwide suffer from Se deficiency. In spite of the important role of Se, its concentrations in soil, water and essential crops have not been studied in Iran. Therefore, the main aim of the current study was to determine the Se content of soil, water, and essential crops (rice in North, wheat in Center, date, and pistachio in South) of different regions of Iran. Sampling was performed in the North, South, and Central regions of Iran. In each selected area in the three regions, 17 samples of surface soil were collected; samples of water and essential crops were also collected at the same sampling points. Upon preliminary preparation of all samples, the Se concentrations were measured by ICP-OES Model Varian Vista-MPX. The amount of soil-Se was found to be in the range between 0.04 and 0.45 ppm in the studied areas; the Se content of soil in the central region of Iran was the highest compared to other regions (p<0.0001). The average Se concentration in irrigation water of different areas was less than 0.01 mg/L, and the mean concentrations of Se in the rice, wheat, date, and pistachio samples were 0.95, 0.74, 0.46, and 0.40 ppm, respectively. Although Se-soil and water-Se level in different regions were low, the typical levels of Se in the essential crops were relatively high.

## Introduction

Selenium (Se), as an essential part of nutrition for human, animal, and many bacteria was first reported in 1957 by Schwarz and Foltz [1]. Se is a rare element in our planet, with the mean concentration of 0.05 mg/kg in igneous bedrock, which is less than any other nutrient element [2]. Excess or insufficient Se intakes can result in adverse effects on human health [3].

Selenium plays an important role in a number of metabolic functions including antioxidant systems, thyroid hormone metabolism, immune function and reproduction. A congestive cardiomyopathy caused by dietary deficiency of Se is called Keshan disease. To the best of our knowledge, it was reported for the first time in 1979 by the Chinese scientists [4].

Selenium range between dietary deficiency and excess is fairly narrow. Diseases such as cancer, cardiomyopathy, myocardial deaths, arthritis rheumatoid, as well as the lesions caused by Failure To Thrive (F.T.T.) are more likely to develop in populations with insufficient exposure to Se [5-10]. On the other hand, excess Se intakes have been shown to be associated with selenosis (pathological changes to the hair and nails), skin lesions, and neurological effects [11].

During the past decade, a growing body of research on humans and animals has revealed the importance of heavy metals for optimum health [12-14]. Thus, dietary recommendations for Se are set up by the World Health Organization (WHO) and Recommended Daily Allowance (RDA) ([11,15]).

Exposure to Se may happen in several different ways including food, water, or even in contact with soil or air that contains high concentrations of Se. However, the uptake of Se by humans mainly takes place from foodstuffs such as grains, cereals, and meat. Se concentrations in foods vary broadly between different types of foods and regions [11]. Despite the significant role of Se in preventing certain degenerative diseases such as cancer and arthritis rheumatoid, the Se contents of soil, water, and the staple food such as rice and wheat produced in Iran have not been evaluated. In addition to the possible link between Se deficiency and degenerative diseases, there is currently an idea that certain viruses such as HIV-Aids and avian flu may interact directly with Se in host cells. Thus, spread of viral diseases can be hastened in the Se-deficient parts of the world [16].

Although Se-soil concentrations of the geographic origin of the crop is the main determinant of the Se content of foods, other factors such as climatic conditions and using Se-rich fertilizers can affect Se concentrations of foodstuffs [17]. There is no national Se-soil geochemistry database for Iran; therefore, the objectives of this study were firstly to identify Se-soil concentrations in different regions of Iran and secondly to investigate the Se contents of soil, water and staple foods in the selected areas.

## Materials and methods

**Figure 1:**
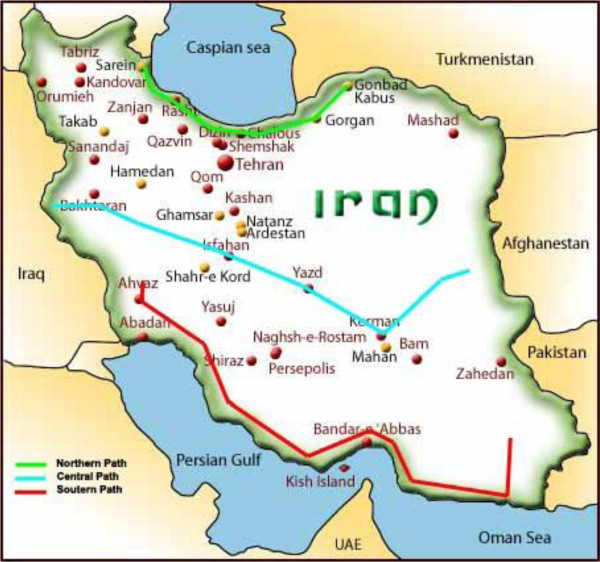
The pathway schema of the selected sampling points in North, South, and Center of Iran.

Sampling was performed from the agricultural fields in the North, South, and Central parts of Iran (from east to west). Using the map of Iran (Figure 1) and specifying the length of sampling route, the researchers computed the dimensions approximately 850 km northward; 2000 km southward, and 2350 km toward the Central region. Sampling was performed in 17 locations in each region of the North, South and Centre (Table 1). Then, the sampling points were determined in the northern region at an approximate distance of 50 km, in the southern region at an approximate distance of 118 km, and in the central region at an approximate distance of 138 km from each other.

**Table 1:** Selected sampling points in North, South, and center of Iran

**Route:**		**North of Iran**	**Center of Iran**	**South of Iran**
Start-up:		Gonbadkavos	Khosf	Dezfool
Termination:		Ardebil - Gheshlaghi	Shabab-Iylam	Iranshahr
**Areas under coverage**	1	Gonbadkavos	Kosf	Dezfoul
	2	Khanbebin	Niyband	Ahvaz
	3	Sorkhankelate	Ravar	Omidiye
	4	Kordkoy	Kerman	Behbahan
	5	Rostamkola-Behshahr	Rafsanjan	Borazjan
	6	Sari area	Anar	Bandar Dayer
	7	F.Kenar-Mahmodabad	Mehriz	Asalooyeh
	8	Nour-chamestan	Yazd	Bandar Charok
	9	Noshahr	Aghda	Bandar Khamir
	10	Abbasabad-Nowshahr	Naeen	Bandar Abbas
	11	Ramsar	Kohpaye	Minab
	12	Langaroud	Najafabad	Bandar Jask
	13	Khomam	Daran	Jahlo - Lyrdof
	14	Rezvanshahr	Azna	Kahir (Birdof)
	15	Lisar	Khoramabad	Chahbahar
	16	Astara-Abbasabad	Kohdasht	Rask
	17	Ardebil-Gheshlaghi	Shabab-Iylam	Iranshahr
**Length of route**		850 km	2350 km	2000 km

In each selected area in the North, South, and Central parts of Iran, 17 samples of surface soil (at a depth of 20 cm), water and essential crop (rice in north, wheat in center, date and pistachio in the south) were collected from agricultural fields at the same sampling points. pH of water samples were immediately justified after collection to < 2 by means of nitric acid (65%). The samples were kept in special sampling vessels, refrigerated, and sent to the research laboratory of the college of Science, Faculty of Chemistry, Tehran University, within 24 hours [18,19].

The samples of polished rice, polished wheat, date and pistachio were ground and dried at 80°C before analysis. One gram of each solid sample was added to a beaker containing a volume of 5 mL HNO_3_ (65%) and 2 mL HClO_4_ and covered with a watch glass; the mixture was then heated to decreasing the volume to 3–5 mL through evaporation. Afterward, 10–15 mL deionized water was added to the solution and then passed through an acid washed paper filter. Finally, the solution was diluted to 50 mL with deionized water in an acid washed volumetric flask.

2–5 mL of deionized water was added to 1g of oven-dried soil samples. Aqua Regia (10 mL) was then added to the solution and sample vessel was covered with a watch glass and heated for 2 hours to digest. After adding 10–15 mL deionized water to the solution, the sample solution was passed through a paper filter and the solution was diluted to 50 mL with deionized water.

Upon preliminary preparation using Aqua Regia digestion method, the Se rates were measured by ICP-OES Model Varian Vista-MPX.

Geographical and regional information such as rainfall, used pesticide, types of irrigation, and elevation of sampling points were collected through a questionnaire. All analyses were undertaken using Minitab (Version 16). A value of p<0.05 was defined as significant.

## Results

**Figure 2:**
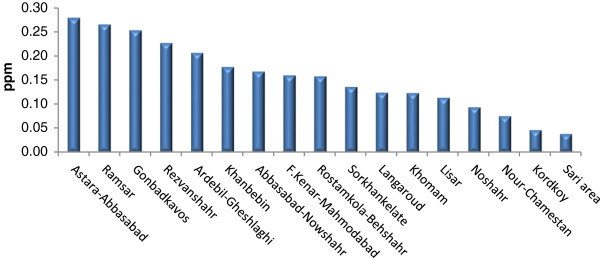
Mean of total soil Se in selected areas of North of Iran.

**Figure 3:**
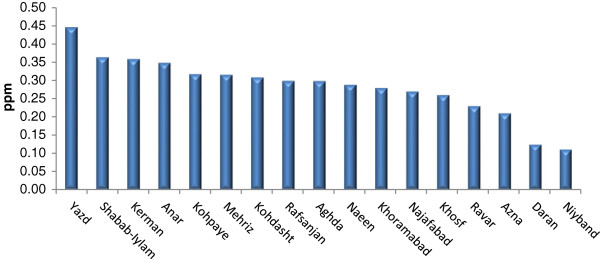
Mean of total Se-soil in selected areas of Center of Iran.

**Figure 4:**
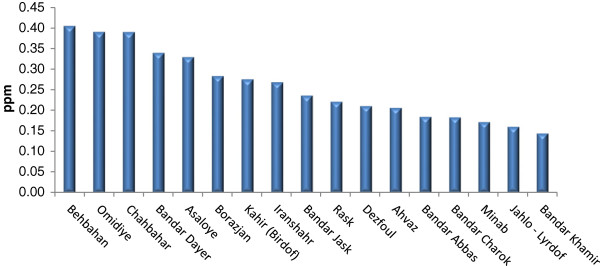
Mean of total Se-soil in selected areas of South of Iran.

Tables 2, 3, and 4 show Se contents found in the soils and essential crops collected from the agricultural lands of North, South, and Center of Iran. Se-soil concentrations in all areas ranged from 0.04 (Sari area) to 0.45 (Yazd area) ppm (Figures 2, 3 and 4). As Table 5 indicates, the average Se-soil level in the Central part of Iran was significantly higher than the North and South (F= 11.922, p<0.0001).

**Table 2:** Selected sampling points and selenium contents of soil and rice as an essential crop in the North of Iran

**Route:**		**North of Iran**		
		**Region**	**Soil-Se (ppm)**	**Rice-Se (ppm)**
**Areas under coverage**	1	Gonbadkavos	0.25	1.49
	2	Khanbebin	0.18	0.91
	3	Sorkhankelate	0.14	0.96
	4	Kordkoy	0.05	0.91
	5	Rostamkola - Behshahr	0.16	0.79
	6	Sari zone	0.04	0.95
	7	F.Kenar-Mahmodabad	0.16	0.62
	8	Nour, chamestan road	0.08	1.31
	9	Noshahr	0.09	1.14
	10	Abbasabad-Nowshahr	0.39	0.79
	11	Ramsar	0.27	0.98
	12	Langaroud	0.12	0.50
	13	Khomam	0.12	0.84
	14	Rezvanshahr	0.23	1.08
	15	Lisar	0.11	1.25
	16	Astara-Abbasabad	0.28	0.74
	17	Ardebil-Gheshlaghi	0.21	0.95
Length of route		850 km		

**Table 3:** Selected sampling points and selenium contents of soil and essential crops in the center of Iran

**Route:**		**Center of Iran**			
		**Region**	**Soil-Se (ppm)**	**Crop type**	**Se content (ppm)**
**Areas under coverage**	1	Khosf	0.26	Wheat	0.99
	2	Niyband	0.11	Date	0.26
	3	Ravar	0.23	Wheat	0.69
	4	Kerman	0.36	Pistachio	0.43
	5	Rafsanjan	0.30	Pistachio	0.32
	6	Anar	0.35	Pistachio	0.46
	7	Mehriz	0.32	Wheat	0.52
	8	Yazd	0.45	Wheat	*NM
	9	Aghda	0.30	Wheat	0.34
	10	Naeen	0.29	Wheat	0.74
	11	Kohpaye	0.32	Wheat	*NM
	12	Najafabad	0.27	Wheat	0.92
	13	Daran	0.12	Wheat	0.51
	14	Azna	0.21	Wheat	1.0
	15	Khoramabad	0.28	Wheat	1.44
	16	Kohdasht	0.31	Wheat	0.50
	17	Shabab-Iylam	0.36	Wheat	0.48
Length of route		2350 km			

*Not Measured.

**Table 4:** Selected sampling points and selenium contents of soil and essential crops in the South of Iran

**Route:**		**South of Iran**			
		**Region**	**Soil-Se (ppm)**	**Crop type**	**Se content (ppm)**
**Areas under coverage**	1	Dezfoul	0.21	Wheat	0.82
	2	Ahvaz	0.21	Wheat	0.79
	3	Omidiye	0.39	Wheat	0.67
	4	Behbahan	0.41	Wheat	0.79
	5	Borazjan	0.28	Wheat	0.54
	6	Bandar Dayer	0.34	Wheat	0.73
	7	Asaloye	0.33	Wheat	*NM
	8	Bandar Charok	0.18	Date	0.57
	9	Bandar Khamir	0.15	Date	0.33
	10	Bandar Abbas	0.19	Date	0.47
	11	Minab	0.17	Date	0.49
	12	Bandar Jask	0.24	Date	0.32
	13	Jahlo - Lyrdof	0.16	Date	*NM
	14	Kahir (Birdof)	0.28	Date	0.60
	15	Chahbahar	0.39	Date	0.53
	16	Rask	0.22	Date	0.36
	17	Iranshahr	0.27	Date	0.62
Length of route		2000 km			

*Not measured.

**Table 5:** Mean soil selenium level (ppm) in different regions of Iran (North, South and Center)

**Regions**	**No. of samples**	**Mean**	**Sd**	**Max**	**Min**	**F**	**P value**
**North**	**17**	0.156	0.018	0.28	0.04	**11.922**	**< 0.0001**
**South**	**17**	0.260	0.021	0.41	0.15		
**Center**	**17**	0.284	0.020	0.45	0.11		

The results did not show statistically significant difference in the mean Se-soil level at different types of irrigation, pesticide exposure, and elevation of sampling points as determined by ANOVA (p>0.05). The soils in the area with high level of rainfall had a significantly lower concentration of Se (p<0.05). However, this significant difference disappeared after adjustment for region (p>0.05).

**Figure 5:**
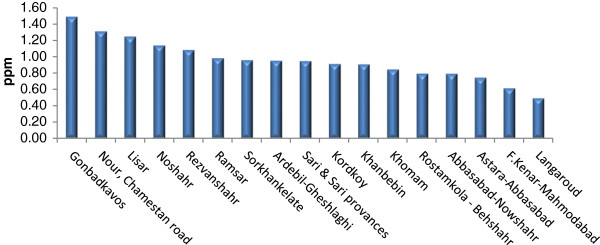
Mean of total Se-Rice in selected areas of North of Iran.

**Figure 6:**
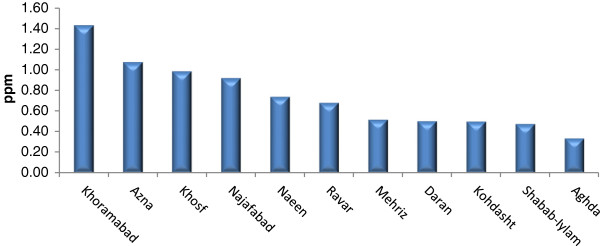
Mean of total Se-wheat in selected areas of Center of Iran.

**Figure 7:**
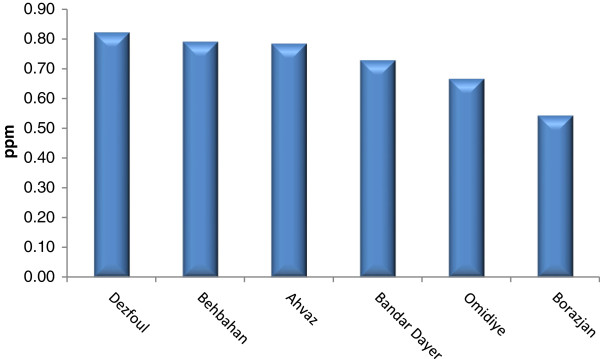
Mean of total Se-wheat in selected areas of South of Iran.

**Figure 8:**
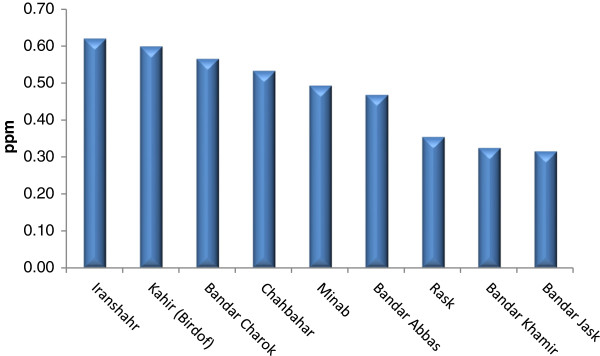
Mean of total Se-date in selected areas of South of Iran.

The average of Se concentrations in rice samples collected from north of Iran ranged from 0.50 ppm (Langaroud) to 1.49 ppm (Gonbadkavos), (Figure 5). The average concentration of Se in wheat samples collected from the center and south of Iran ranged from 0.34 ppm (Aghda) to 1.44 ppm (Khoramabad) and 0.54 ppm (Borazjan) to 0.82 ppm (Dezfoul), respectively (Figures 6, 7). Figure 8 shows the average Se concentration in date in selected areas of south of Iran. Maximum and minimum concentrations of Se were 0.62 ppm (Iranshahr) and 0.32 ppm (Bandar Jusk), respectively.

The average, maximum and minimum Se concentrations obtained in the rice, wheat, date, and pistachio samples are given in Table 6. The results of this study showed that, selenium concentration in the irrigation water of all sample points were less than 0.010 mg/L (Table 7).

**Table 6:** Mean, maximum, minimum and standard deviation of Se content (ppm) of essential corps (rice, wheat, date, and pistachio) in different regions of Iran (North, South and Center)

**Type of sample**	**No. of samples**	**Mean**	**Sd**	**Max**	**Min**	**F**	**P value**
Rice (North)	17	0.95	0.25	1.46	0.50	12.039	<0.0001
Wheat (Center & South)	17	0.74	0.27	1.44	0.34		
Date (Center & South)	10	0.46	0.13	0.62	0.26		
Pistachio (Center)	3	0.40	0.74	0.46	0.32		

**Table 7:** Water selenium level (mg/L) in selected sample points of Iran (detection limit=0.010 mg/L)

**Sample name**	**Se concentration (mg/L)**
North areas (all samples)	< 0.010
Central areas (all samples)	< 0.010
South areas (all samples)	< 0.010

## Discussion

The results of preset study indicated that the average geographical distribution of Se-soil (0.23 ppm) is below the worldwide average (0.400 ppm) reported previously [20]. The Se concentrations of the soils were generally low and much lower than the recommended threshold (0.6 ppm) for grazing livestock [20].

The average Se-soil concentrations obtained are markedly lower than the values in the Se-rich areas of the world such as the Western United States including all or parts of the states of Montana, South Dakota, Wyoming, Utah, Nevada, California, Arizona, and New Mexico, but clearly higher than the low-Se areas such as China, Siberia, Central Africa, Eastern Europe, New Zealand, Russia, and Finland [21,22]. However, it should be noticed that the rate of Se in soil varies widely around the world and in most parts of the world, total Se-soil concentrations are about or lower than 0.1 to 2 ppm [16].

The results demonstrated that the average Se-soil concentrations are significantly different among the selected areas of North, South and Center of Iran. The average total Se-soil concentration in the Center of Iran was greater than that in the North and South (Table 5). A number of factors such as soil geological parent materials, rainfall, pesticide exposure, types of irrigation, and elevation of sampling points can determine Se concentrations of soils [16]. No significant correlations were observed between total Se-soil concentration and the studied variables including pesticide exposure, types of irrigation, and elevation of sampling points. The results of the current study showed that although soil in the area with high level of rainfall had a significantly lower concentration of Se, this significant difference disappeared after adjustment for region. This finding may reflect the importance of geological parent materials in Se concentration of soil.

Determining the concentration of Se in the essential crops, which are the major dietary sources of Se in most countries including Iran, was the second aim of this study. Rice is the main product of farmers in the north, and wheat and date are the major agricultural products in the south of Iran. The main products in the central lands of Iran are wheat, date, and pistachio. These crops except pistachio are somehow considered as staple foods for Iranian population in each region, therefore Se content of these foods has a great influence on their overall dietary Se intake. Similar to Se-soil content, the Se concentration in cereal grains was also very variable. Clearly, the Se content of cereal grains depends on the amount of Se available in soils, particularly when grown in alkaline soils (Kabata-Pendias and Pendias, 1994; [23]).

The Se content of the rice samples ranged from 0.5 to 1.46 ppm, which was quite higher than the results reported for the average Se content of rice which were 0.05 ppm in Thailand, 0.02 ppm in China, 0.073 ppm in New Zealand, 0.319 ppm in USA, and 0.1 ppm in UK [24-26]. Despite the low Se-soil levels in the north of Iran (0.156 ppm), the average level of Se in rice was high (0.95 ppm). This is because of bioavailability of selenium forms. Selenate is less strongly adsorbed to minerals in the soil and more readily taken up by plants than selenite. Many factors such as dry climate, low organic matter concentration in the soil, high temperature and pH, and no water-logging may give a high ratio between selenate and selenite in the soil. Selenite, however, is the dominant form of inorganic Se in soils with high concentrations of organic matter, as in the Nordic countries (because of low soil temperatures causing much slower degradation of soil organic matter than in tropical countries), and most likely also in waterlogged soils (during rice cultivation). Still, further studies are needed to determine the role of other factors that contribute to this level of Se in rice. In addition, rice is a staple food in Iran particularly in the North; therefore, it would be particularly interesting to see if there is a correlation between Se contents of rice and serum Se in the north of Iran.

The wheat samples were found to contain 0.34-1.44 ppm with an average of 0.74 ppm of Se (Table 6). These values are higher than the reported Se concentration range of 0.02-0.60 ppm for most of the world’s wheat [27]. The average value is 10 times higher than those previously reported for Se in the wheat grain samples collected from different parts of Saudi Arabia. The most extreme values (up to 30 ppm) for the Se concentrations of wheat have been found in the seleniferous areas of China and India [28]. Values ranging from 0.009 to 0.034 ppm have been reported for Sweden, Germany, Scotland, and Norway [29]. Variation of metal concentrations in the soils will result variation in elemental uptake by parts of plants [30]. The Se concentration of the wheat samples in this study was higher than the values reported for the seleniferous areas of Venezuela which were found to be in the range of 0.025 to 0.250 ppm [31].

Dry or soft dates are consumed as a staple of Mediterranean diets for millennia. This is due partially to its original cultivation countries such as Iraq, Iran, Syria, southwest Turkey, and northern Egypt. Literature review shows that date originally belongs to the area of the Persian Gulf. Another reason could be the nutritional values of date which is an important source of vitamins, minerals and fiber. To our knowledge, only one study has so far determined the Se content of date [32]. The average Se concentration of the date in the present study was markedly lower than those reported for Saudi Arabia [32] (Table 6). In some areas of Saudi Arabia, Se content in the date was found to be in the range of 1.48 to 2.96 ppm [32]. The Se content of date samples from the areas studied ranged from 0.26 to 0.62 ppm, with an average of 0.46 ppm. The observed Se content of the date samples was higher than the value (0.03 ppm) in USDA nutrient database.

Iran is the greatest pistachio producer and exporter in the world with a production of more than 190,000 tons per year. The amount of Se in the pistachio samples collected from the South regions of Iran was found to be in the range between 0.32 and 0.46 ppm, with an average of 0.40 ppm (Table 6). When these data is compared with the USDA information, the average Se content of our samples was higher than the USDA values (0.07 ppm).

Natural background concentrations of Se in freshwater usually ranges from 0.1-0.4 ppb [33]. Nevertheless, the Se levels in all collected samples of the irrigation water in different areas of Iran were less than 0.01 ppm (Table 7), showing that the Se content of water in Iran is lower than the regular range.

Despite the low levels of Se in Iran’s soils and water samples, the average Se concentrations of essential crops including wheat, rice, date, and pistachio were quite high. Further studies are needed to identify the effect of potential factors such as soil pH, redox potential, calcium carbonate level, cation exchange capacity, organic carbon, iron and aluminum levels on cereal potential to accumulate Se.

## Competing interests

The authors declare that they have no competing interest.

## Authors’ contributions

LN, has intellectual property, written the original research plan of project and wrote the manuscript. SN, implemented the sampling framework, supervised the methods of analyses and wrote the manuscript. MRE implemented the statistical analysis framework and analyzed the datasets. SN, supervised and edited the Manuscript. KD, wrote the manuscript. MY, supervised the statistical aspects of the work. HS, Analysed the Samples. AM, participate in determination of sample points in Se map of Iran. SJS, in process of sample preparation procedure and edited the Manuscript. All authors read and approved the final manuscript.
